# Alteration of the Rhizosphere Microbiota and Growth Performance of Barley Infected with *Fusarium graminearum* and Screening of an Antagonistic Bacterial Strain (*Bacillus amyloliquefaciens*)

**DOI:** 10.3390/microorganisms13051010

**Published:** 2025-04-27

**Authors:** Yang Fu, Jing Luan, Jialei Shi, Wenzhu Tang, Xianzhen Li, Zhimin Yu, Fan Yang

**Affiliations:** School of Biological Engineering, Dalian Polytechnic University, Dalian 116034, China; fuyang202006@163.com (Y.F.); luanjing426@163.com (J.L.); shijialei1998@163.com (J.S.); tangwz@dlpu.edu.cn (W.T.); xianzhen@mail.com (X.L.)

**Keywords:** microbiota transplant, barley seedling, rhizosphere, bacterial community

## Abstract

*Fusarium graminearum* is one of the most important pathogenic fungi with a wide range of plant and animal hosts. This study investigated the effects of *F. graminearum* infection on the rhizosphere microbiota and growth of two barley (*Hordeum vulgare* L.) cultivars, Baudin and Kenpi 7, and explored microbiota transplantation as a strategy to enhance disease resistance. By exchanging surface microbiotas between varieties and analyzing rhizosphere bacterial communities using 16S rRNA sequencing, researchers observed that *F. graminearum* infection increased bacterial diversity and abundance, especially in Baudin barley. Growth indicators (root length, plant height, fresh/dry mass) also exhibited that Baudin barley showed stronger resistance. Functional analysis underscored that the microbial community composition of Baudin barley promoted metabolic pathways related to plant resilience and was associated with improved seedling health. In contrast, Kenpi 7 barley showed weaker resistance, emphasizing the role of seed-specific microbiotas in pathogen defense. An effective antagonistic strain, *Bacillus amyloliquefaciens* B1, was isolated from Baudin barley, and its inhibition rate against *F. graminearum* was 80%. The results showed that microbiota transplantation enhanced the disease resistance of low-diversity seeds, and identified *B. amyloliquefaciens* B1 as a promising biocontrol agent, providing a potential application for sustainable agriculture and reducing dependence on chemical fungicides. This study highlights the importance of seed-associated microbial communities in plant–pathogen interactions and provides a basis for the development of microbiota-based strategies to mitigate crop diseases.

## 1. Introduction

*Fusarium graminearum* can cause disease in many important economic crops, including maize (*Zea mays* L.), wheat (*Triticum aestivum* L.), and barley (*Hordeum vulgare* L.) [1]. Barley is one of the main crops for food and beverage production globally and is widely cultivated worldwide [2]. The plant can be infected by *F. graminearum* at all stages, causing two epidemic diseases of root rot and *F. graminearum* head blight, which seriously affect grain yield and quality [3]. *Fusarium* is also a producer of multiple mycotoxins and is considered to be an opportunistic human pathogen [4]. To effectively control plant diseases and reduce grain losses, many fungicides that can control the growth of *Fusarium* have been developed [5]. Commercial preservatives (such as sodium propionate and sodium benzoate) and chemical fungicides (such as prochloraz and metalaxyl) have been widely used to control plant pathogens [6].

In order to reduce fungicide residues and protect land sustainability, biological control agents have been a research hotspot in recent years. Several studies have reported the positive effects of beneficial bacteria on several plant diseases, and commercial products have been sold [7]. Beneficial microbes are considered important alternatives to chemical plant protection strategies for the improvement of plant performance and health in agricultural systems [8]. Plant-associated microbes are known to have important roles in plant health and disease [9]. The major antagonistic bacteria, including *Bacillus*, *Pseudomonas*, *Trichoderma*, *Lactobacillus*, and *Streptomyces*, have been shown to effectively control pathogenic fungi and reduce the incidence and severity of diseases [10].

However, most of the research is mainly based on plant rhizosphere soil, and few studies have described the role of seed bacterial communities. Plant seed microorganisms can preserve and spread plant-specific core microbiota [11]. Seed microbes can colonize before seedling roots recruit microbes from the surrounding environment and promote germination and early survival of plants [12]. Therefore, the seed bacterial community may be used to inhibit pathogenic fungi. In order to prove the antagonistic reaction of the microbial community of barley seeds to *Fusarium*, we selected Baudin barley and Kenpi 7 barley from two different production areas for experiments. We exchanged the surface microbiome of two barley varieties to achieve the conditions of different surface microbiota of the same variety of barley. Then barley seedlings were cultured, the growth of barley seedlings was measured and the rhizosphere bacterial microbiota was analyzed using 16S rRNA sequencing. We elucidated microbiota-mediated resistance mechanisms by linking microbial diversity shifts to plant growth metrics, identified keystone taxa contributing to *Fusarium* suppression, and isolated and characterized novel biocontrol agents from seed microbiotas. This approach diverges from traditional soil-focused biocontrol studies by emphasizing seed microbiotas as a direct, heritable source of plant protection. Furthermore, our integration of 16S rRNA sequencing, functional pathway analysis (FAPROTAX), and in vitro antagonism assays provides a multi-dimensional perspective on microbiota–pathogen interactions. The discovery of *Bacillus amyloliquefaciens* B1 demonstrates the untapped potential of seed microbiotas in developing targeted biocontrol solutions. Our findings underscore the importance of preserving and harnessing seed microbial diversity as a frontline defense against crop diseases.

## 2. Materials and Methods

### 2.1. Microorganisms and Materials

This study used two barley varieties from different production areas and growth environments. One was Kenpi 7 barley produced in northeast China, and the other was Baudin barley from South Australia.

The plant-pathogenic fungi, *F. graminearum*, were cultured in the laboratory of the School of Bioengineering, Dalian Polytechnic University. Potato dextrose agar (PDA) was inoculated with *F. graminearum* and cultured at 30 °C for 7 days. Bacteria were cultured on Luria–Bertani (LB) medium at 30 °C.

### 2.2. Preparation of Surface Microbiota Exchange

The surface of barley was disinfected, and 30 g of barley was weighed, soaked in 3% H_2_O_2_ for 20 min, washed with sterile water 5 times, and irradiated with ultraviolet light for 30 min to obtain surface-sterile barley [13]. The 200 µL final washing solution was inoculated on the LB plate to test the sterility of the barley surface.

For the barley surface microbiota extraction, we added 30 g of barley to a conical flask with 100 mL of sterile physiological saline (0.9% *w*/*v* NaCl). We oscillated the mixture at 200 rpm for 30 min at room temperature, and then collected the soaking liquid of the barley for later use [14].

**Figure 1:**
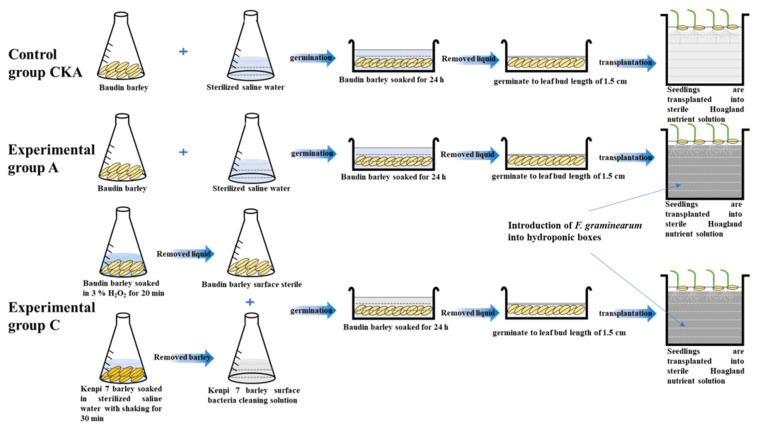
The experimental design of barley hydroponics. In the control group and the experimental group CK A/A, Baudin barley was soaked in sterile water for 24 h. In the experimental group C, Baudin barley was disinfected with H_2_O_2_. The surface microbiota were extracted from Kenpi 7 barley with sterile water, and then the extract was added to the disinfected Baudin barley and soaked for 24 h. After wet soaking, the solution was removed and the seeds were germinated at 16 °C and 90% humidity. When the germinating seeds reached 1.5 cm in length, they were transplanted into a hydroponic box. The seedlings were grown at room temperature in a sterile Hoagland nutrient solution. The experimental groups A and C were inoculated with *Fusarium* solution in a hydroponic box.

The process of exchanging microbiota between the surfaces of the two barley varieties is shown in Figure 1. The soaking liquid of Kenpi 7 barley was added to the aseptically treated Baudin barley and placed in a plant incubator at 16 °C for 24 h [15]. After the soaking solution was removed, the barley germinated to 1.5 cm long at a humidity of 90% and 16 °C. It was then taken out of the incubator and kept on standby; this group was denoted as experimental group C [16]. The same method was used to inoculate the surface microbiota of Baudin barley onto the disinfected Kenpi 7 barley.

The grouping, treatment and numbering in the barley seedling hydroponic experiment were as follows. CKA, A, C (Baudin barley): CKA—control group not inoculated with *F. graminearum*, own microbiota; A—experimental group inoculated with *F. graminearum*, own microbiota; and C—experimental group inoculated with *F. graminearum*, and inoculated with surface microbiota of Kenpi 7 barley after disinfection. CKB, B, D (Kenpi 7 barley): CKB—control group not inoculated with *F. graminearum*, own microbiota; D—experimental group inoculated with *F. graminearum*, own microbiota; D—experimental group, inoculated with *F. graminearum*, inoculated with surface microbiota of Baudin barley after disinfection.

### 2.3. Barley Seedling Culture

First, the barley seeds were germinated. Baudin barley (30 g) and Kenpi 7 barley (30 g) were soaked in 100 mL of sterile water in a plant incubator at 16 °C for 24 h. The seeds were then removed from the water and allowed to germinate and grow to a leaf bud length of 1.5 cm in 90% humidity and 16 °C before removal for later use.

Then, the barley seedlings were transplanted into hydroponic boxes, and 800 mL of sterilized Hoagland nutrient solution was added to each hydroponic box. The experimental group was inoculated with *F. graminearum* in nutrient solution (inoculation amount 0.1%, spore concentration 10^7^ CFU/mL) and cultured at room temperature for 10 days.

### 2.4. Barley Rhizosphere Microbiota Sample Preparation

At the end of the hydroponic growth period, 20 barley plants were taken from each experimental group, A, B, C, and D, and the control groups, CKA and CKB. The root part was cut off and placed in a 50 mL centrifuge tube. Then, 25 mL sterile water was added and the mixture thoroughly shaken. The supernatant was then removed and stored at −80 °C.

### 2.5. Total Community DNA Extraction and 16S rRNA Gene Amplicon Library Preparation

Total bacterial DNA was isolated from samples using the FastDNA™ SPIN Kit for Soil (MP Biomedical LLC, Santa Ana, CA, USA) according to the manufacturer’s protocol. The concentration and purity of the metagenomic DNA extracted were measured using a spectrophotometer (Invitrogen Qubit 3.0, Thermo Fisher Scientific, Waltham, MA, USA). The V3–V4 region of the bacterial 16S rRNA gene was amplified by polymerase chain reaction with the common primer pair 341 F (5′-CCTACGGGNGGCWGCAG-3′) and 785R (5′-GACTACHVGGGTATCTAATCC-3′) combined with adapter sequences and barcode sequences. High-throughput sequencing analysis of bacterial rRNA genes was performed on the Illumina NovaSeq platform (2 × 250 paired ends) at Genesky Biotechnologies Inc. (Shanghai, China).

### 2.6. Effect of Exchange Rhizosphere Microbiota on the Growth of Barley Infected with Fusarium

After 10 days of barley hydroponics, 10 barley seedlings were taken from the hydroponic box to measure root length, plant height, dry mass, fresh mass, and root vigor (TTC method) [17,18].

### 2.7. Isolation of Fusarium Antagonistic Bacteria from the Surface of Barley Seeds

The Baudin barley was suspended in sterile distilled water, and the resuspension was diluted in a gradient of 10^−4^, 10^−5^, and 10^−6^. The diluted liquid was spread on an LB solid medium and cultured at 30 °C. Single colonies were picked and transferred to an LB solid medium and incubated at 30 °C. After colony growth, the previous steps were repeated until a pure culture was obtained [19].

### 2.8. Screening of Fusarium Antagonistic Bacteria

Screening of strains that can improve the resistance of barley to root rot was performed. Based on the microorganisms isolated as described in Section 2.7, the antagonistic activities of the active bacteria against *F. graminearum* were evaluated. PDA plates and filter paper discs (round, 5 mm in diameter, sterilized) were used for antagonistic experiments. The filter paper disc was placed in the center of the PDA plate, and 3-day-old *F. graminearum* fermentation broth was added. Four filter paper discs were placed at a distance of 2 cm from the center of the PDA plate, and the bacterial fermentation broth to be evaluated was inoculated (repeated three times). Specifically, a filter paper disc with a diameter of 5 mm containing 10 µL 10^6^ CFU/mL bacterial fermentation broth and 10 µL 10^6^ spores/mL fungal fermentation broth was used for antagonism. After 5 days, the diameter and inhibition rate of the inhibition zone were evaluated.

### 2.9. Hydrolytic Enzyme Activity of Bacteria on Barley Surface

The strains with antibacterial activity were fermented in LB medium (30 °C, 200 rpm) for 3 days and centrifuged at 8000 rpm/min for 20 min at 4 °C. The supernatant was taken to determine the activity of protease, β-glucanase, chitinase, and xylanase according to the reported method [20,21,22]. Each experiment was repeated three times. The bioassay data were expressed as the mean of three replicates.

### 2.10. Identification of Effective Antibacterial Strains

The DNA of the antifungal strain was extracted using a Bacterial Genomic DNA Extraction Kit (Shenggong, Beijing, China) in accordance with the manufacturer’s instructions. The *16S rRNA* and *gyrA* genes sequences fragment of strain B1 was amplified using the primer pair of 27F (5′-AGAGTTTGATCCTGGCTCA-3′)/1492R (5′-GGTTACCTTGTTACGACTT-3′) and GyrA-F (5′-CAGTCAGGAAATGCGTACGTCCTT-3′)/GyrA-R (5′-CAAGGTAATGCTCCAGGCATTGCT-3′). All PCR reactions were performed in a total volume of 50 µL. PCR was performed with a T100™ Thermal Cycler (Bio-Rad, Hercules, CA, USA) with pre-deformation at 94 °C for 5 min, 30 cycles of denaturation for 1 min at 94 °C, annealing for 1 min at 55 °C, and extension for 90 s at 72 °C, followed by a final step of 72 °C for 5 min.

The amplified products were sequenced (BGI, Beijing, China). The *16S rRNA* and *gyrA* genes sequence of the antifungal strain were subjected to the NCBI BLAST query, and Phylogenetic trees were constructed with the neighbor-joining method using MEGA 11.

### 2.11. Analysis of Antibacterial Components of Strain B1

The antibacterial activity of the cell content extract (CC) of strain B1, the sterile fermentation liquid (SF) of strain B1, and the volatile compound (VOC) produced by strain B1 were determined, and the antibacterial components of strain B1 were preliminarily determined. Referring to the method described in Section 2.8, The sample to be tested was coated on a PDA plate, and the *Fusarium* was inoculated on the filter paper disc in the middle of the plate. The antibacterial activity of volatile substances of strain B1 was determined by the two-separated plate method. The two filter paper discs were placed on both sides of the separation plate. Then the fermentation broths of *F. graminearum* and strain B1 were inoculated onto the filter paper discs and cultured at 30 °C for 5 days.

### 2.12. In Vitro Inhibition of Strain B1 on F. graminearum

Baudin barley (30 g) was placed in a 250 mL Erlenmeyer flask and sterilized at 121 °C for 20 min. The autoclaved barley medium was inoculated with 10 µL of strain B1 fermentation broth, 10 µL of 5-day-old *Fusarium* broth and 10 µL of strain B1 fermentation broth, and 10 µL of 5-day-old *Fusarium* broth, respectively. The blank group was not inoculated with anything, and all samples were cultured at 30 °C for 5 days.

### 2.13. Statistical Analysis

All experiments were performed at least 3 times independently and plotted using Origin 2019 software. One-way analysis of variance and Pearson correlation analysis were performed using IBM SPSS Statistics V27 software. Statistical significance was set at *p* < 0.05.

## 3. Results

### 3.1. Effect of F. graminearum Infection on Barley Seedlings

**Figure 2:**
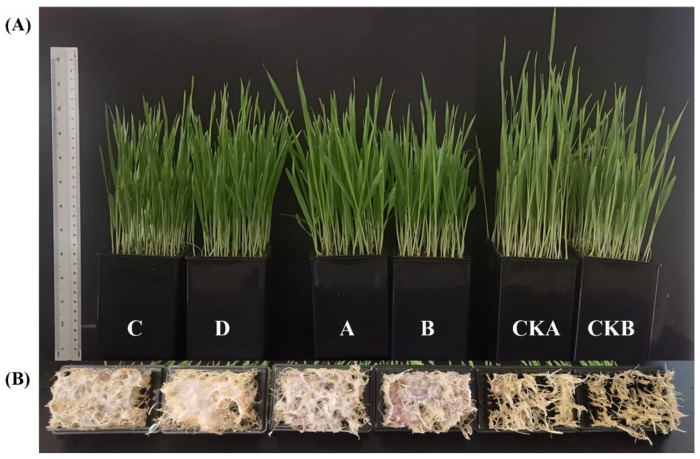
Effects of *F. graminearum* infection on the growth of hydroponic barley seedlings. (**A**) Stem of barley seedlings cultured for 10 days. (**B**) Roots of barley seedlings cultured for 10 days. CKA, A, C (Baudin barley): CKA—control group, not inoculated with *F. graminearum*, own microbiota; A—experimental group, inoculated with *F. graminearum*, own microbiota; C—experimental group, inoculated with *F. graminearum*, and inoculated with the surface microbiota of Kenpi 7 barley after disinfection. CKB, B, D (Kenpi 7 barley): CKB—control group, not inoculated with *F. graminearum*, own microbiota; D—experimental group, inoculated with *F. graminearum*, own microbiota; D—experimental group inoculated with *F. graminearum*, and inoculated with the surface microbiota of Baudin barley after disinfection.

After infection with *F. graminearum*, the plant height of two kinds of barley seedlings was significantly lower than that of healthy barley seedlings (*p* < 0.05), as shown in Figure 2. Moreover, we can see that the roots of barley seedlings infected with *F. graminearum* showed yellowing and wilting.

After infection with *F. graminearum*, the growth indexes of the two varieties of barley seedlings were significantly lower than those of healthy barley seedlings (Table 1). The indexes of barley seedlings in group D were better than those in group B due to the inoculation of the Baudin barley surface microbiota. In comparison, the indexes of barley seedlings in group C were lower than those in group A due to the inoculation of the Kenpi 7 barley surface microbiota. Therefore, the antagonistic effect of the surface microbiota of Baudin barley on *F. graminearum* was higher than that of Kenpi 7 barley. The microbiota on the surface of seeds has resistance to *Fusarium*. The surface microbiota of barley seeds has a specific regulatory ability after the invasion of pathogenic fungi. The excellent microbial community has a more substantial inhibitory effect on pathogens and can improve the disease resistance of plants.

### 3.2. Analysis of Bacterial Communities Diversity in Barley Rhizosphere

**Figure 3:**
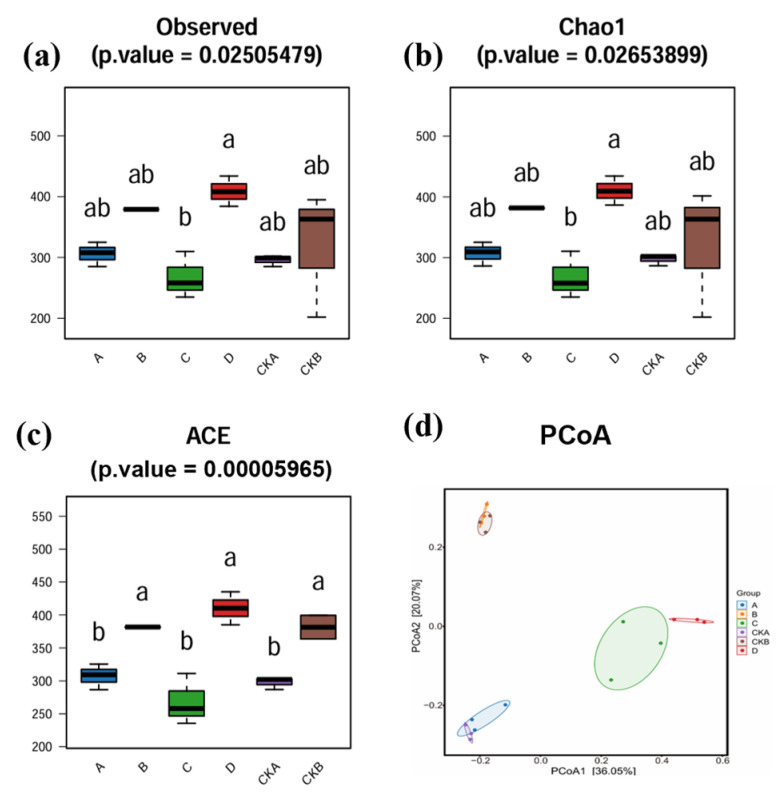
Analysis of bacterial community diversity in barley rhizosphere. Alpha diversity of barley rhizosphere microbial community: (**a**) Observed index, (**b**) Chao1 index, (**c**) ACE index. Statistical analysis was performed by paired Wilcoxon rank sum test. Different letters represent significant differences between groups (*p* < 0.05). The data are expressed as SD ± median (*n* = 3). (**d**) PCoA analysis of the bacterial community in barley rhizosphere based on OUT level and Unifrac distance. CKA, A, C (Baudin barley) CKA—control group, not inoculated with *F. graminearum*, own microbiota; A—experimental group, inoculated with *F. graminearum*, own microbiota; C—experimental group, inoculated with *F. graminearum*, inoculated with the surface microbiota of Kenpi 7 barley after disinfection. CKB, B, D (Kenpi 7 barley): CKB—control group, not inoculated with *F. graminearum*, own microbiota; D—experimental group, inoculated with *F. graminearum*, own microbiota; D—experimental group inoculated with *F. graminearum*, inoculated with surface microbiota of Baudin barley after disinfection.

Analysis of the α-diversity of the barley rhizosphere bacterial community revealed that the rhizosphere bacterial community abundance and diversity of both barley species increased after infection with *F. graminearum*. It can be seen that for the Observed, ACE, and Chao 1 indexes, group A had higher values than the CKA group, and the values for group B were higher than those of the CKB group (Figure 3a–c). After the *Fusarium* infection, the number of OTUs in both barleys increased, and the bacterial community on the barley surface had a positive response after *Fusarium* invasion. The Observed, ACE, and Chao 1 indexes of the C group inoculated with Kenpi 7 barley microbiota were lower than those of the CKA and A groups, and the values of the D group inoculated with Baudin barley microbiota were higher than those of the CKB and D groups. Therefore, the Baudin barley microbiota has more advantages than the Kenpi 7 barley microbiota in antagonistic *F. graminearum*. These results are consistent with the hydroponic growth index of barley seedlings in each group.

Principal coordinate analysis (PCoA) was used to analyze the differences in microbial diversity between groups (Figure 3d). Comparisons between the CKA and A groups, the C and D groups and the CKB and B groups indicate that the C and D groups were significantly different from the other groups in bacterial community diversity. The distribution of parallel samples between groups was similar, indicating that the parallelism between groups was good. The PCoA1 value was 36.05%, and the PCoA2 value was 20.07%, which means that these two axes explained 56.12% of the difference in diversity.

### 3.3. Bacterial Community Composition of Barley Rhizosphere

Amplicon sequencing libraries of barley rhizosphere samples were prepared in a two-step PCR targeting the V3–V4 region of the *16S rRNA* gene. A total of 1470 Operational Taxonomic Units (OTUs) were identified under the 97% sequence similarity threshold, belonging to 12 phyla, 21 classes, 40 orders, 79 families, and 178 genera.

**Figure 4:**
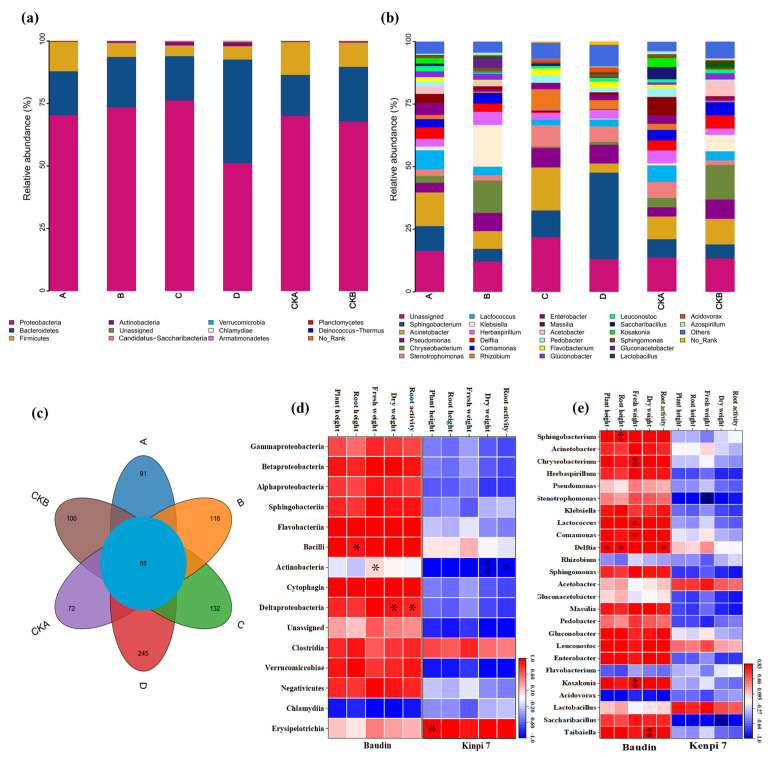
Bacterial community composition in Baudin barley and Kenpi 7 barley rhizosphere under different treatments. (**a**) The relative abundance of rhizosphere bacteria community in the top 10 orders at phylum level. (**b**) The relative abundance of rhizosphere bacteria community in the top 28 orders at genus level. (**c**) OTU distribution comparison Venn diagram. (**d**) Pearson correlation analysis between the abundance of the top 15 orders of rhizosphere bacterial communities at the class level and the growth indexes of barley seedlings. (**e**) Pearson correlation analysis between the abundance of the top 25 orders of rhizosphere bacterial communities at the genus level and the growth indexes of barley seedlings. Pearson correlation analysis was performed using SPSS 27 software. Red indicates a positive correlation, and blue indicates a negative correlation; * means *p* < 0.05, ** means *p* < 0.01. CKA, A, C (Baudin barley): CKA—control group, not inoculated with *F. graminearum*, own microbiota; A—experimental group, inoculated with *F. graminearum*, own microbiota; C—experimental group, inoculated with *F. graminearum*, inoculated with the surface microbiota of Kenpi 7 barley after disinfection. CKB, B, D (Kenpi 7 barley): CKB—control group, not inoculated with *F. graminearum*, own microbiota; D—experimental group, inoculated with *F. graminearum*, own microbiota; D—experimental group inoculated with *F. graminearum*, inoculated with surface microbiota of Baudin barley after disinfection.

In all of the samples analyzed, there were significant differences in the bacterial community structure of barley rhizosphere before and after *F. graminearum* infection. Among the 25 dominant genera we detected, the relative abundances of 7 genera were significantly different before and after *Fusarium* infection (Figure 4).

Proteobacteria, Bacteroidetes, and Firmicutes were the three most dominant phyla, which accounted for a large proportion of the six groups of samples. A total of 34 genera were detected in the samples, of which the dominant genera were *Sphingobacterium*, *Acinetobacter*, *Staphylococcus*, *Spirobacterium*, *Pseudomonas*, *Stenotrophomonas*, *Klebsiella*, *Lactococcus*, *Comamonas*, *Delftia*, *Rhizobium*, *Sphingomonas*, *Acetobacter*, *Gluconacetobacter*, *Marseille*, *Site Bacillus*, *Gluconobacter*, *Candida albicans*, *Flavobacterium*, *Enterobacter*, *Kocuria*, *Acidophilus*, *Lactobacillus*, *Bacillus*, and *Taibai*, a total of 25 (Figure 4a,b). Among them, Kenpi 7 barley had more endemic genera in the rhizosphere flora, and Baudin barley had more species. The abundance of the bacteria increased after the two barleys were infected with *F. graminearum*; the number of species on Baudin barley increased by 12%, and the number on Kenpi 7 barley increased by 8% (Figure 4c). According to Pearson correlation analysis, the abundance of the bacterial community in the barley rhizosphere was positively correlated with the growth index of barley seedlings (Figure 4d,e).

### 3.4. Analysis of Significantly Different Species and Potential Functions of Bacterial Communities in Barley Rhizosphere

**Figure 5:**
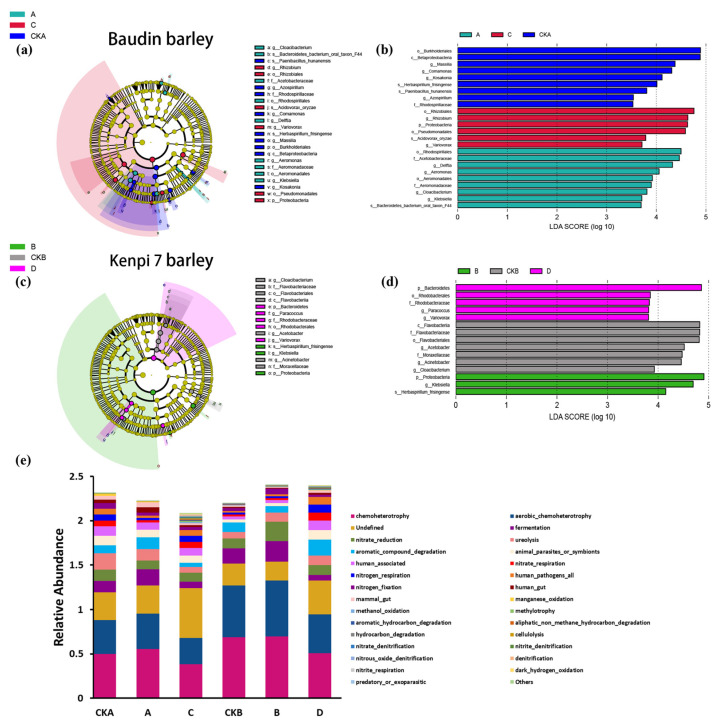
The evolutionary branch diagram of LEfSe analysis of rhizosphere bacterial communities between different treatments for (**a**) Baudin barley and (**c**) Kenpi 7 barley. Linear discriminant analysis (LDA) scores for (**b**) Baudin barley and (**d**) Kenpi 7 barley (only taxa with LDA values greater than 3.5 (*p* < 0.05) are shown). (**e**) FAPROTAX functional subclass histogram. CKA, A, C (Baudin barley): CKA—control group, not inoculated with *F. graminearum*, own microbiota; A—experimental group, inoculated with *F. graminearum*, own microbiota; C—experimental group, inoculated with *F. graminearum*, inoculated with surface microbiota of Kenpi 7 barley after disinfection. CKB, B, D (Kenpi 7 barley): CKB—control group, not inoculated with *F. graminearum*, own microbiota; D—experimental group, inoculated with *F. graminearum*, own microbiota; D—experimental group, inoculated with *F. graminearum*, inoculated with surface microbiota of Baudin barley after disinfection.

The cladogram generated by the LEfSe analysis revealed significant differences in microbial composition between the sample groups (Figure 5a,c). LEfSe analysis showed that in Baudin barley, using a logarithm (LDA) value of 3.5, there were 24 bacterial groups different between A, C, and CKA. Group A was enriched in *Bacteroidetes* (LDA = 4.2), whereas Group C showed dominance in Proteobacteria (LDA = 4.8). The CKA group uniquely harbored plant-growth-promoting taxa like *Azospirillum* (LDA = 4.9) (Figure 5b). LEfSe analysis showed that in Kenpi 7 barley, using a logarithm (LDA) value of 3.5, there were 15 bacterial groups different between B, D, and CKB. This revealed distinct taxonomic biomarkers among groups (Figure 5d). Group B was enriched in *Proteobacteria* (e.g., *Klebsiella*, LDA = 4.0) and *Herbaspirillum frisingense* (LDA = 3.2), while Group D showed a dominance of Bacteroidetes (LDA = 5.0) and *Rhodobacterales*-associated taxa. The CKB group exhibited intermediate features, including *Cloacibacterium* (LDA = 3.5). These results further indicated that *F. graminearum* infection significantly affected the rhizosphere community structure of Baudin barley and Kenpi 7 barley, which was characterized by significant differences in the abundance of dominant groups.

According to the results of the FAPROTAX functional abundance bar chart, 30 bacterial metabolic pathways were found in the rhizosphere of Baudin barley and Kenpi7 barley, such as chemo heterotrophy, nitrate reduction, aromatic compound degradation, nitrogen respiration, nitrogen, and fixation (Figure 5e). The functional abundance of rhizosphere microorganisms of the two barley varieties changed after *F. graminearum* infection. The abundance of nitrogen cycle-related functions (such as nitrate reduction, nitrogen respiration, denitrification, etc.) in the experimental groups (A, C, B, D) was generally higher than that in the control groups (CKA, CKB), especially the abundance of nitrate ddenitrification and nitrogen respiration was significantly increased. The abundance of carbon degradation-related functions (such as aromatic compound degradation, hydrocarbon degradation, etc.) in the experimental group also increased, among which aromatic hydrocarbon degradation and cellulolysis increased significantly. At the same time, there were some differences in the functional abundance of rhizosphere microorganisms between the two barleys after the infection of *F. graminearum*. The experimental group A of Baudin barley was more inclined to have enhanced aromatic carbon degradation and nitrate reduction, while the experimental group B of Kenpi 7 barley showed stronger denitrification and aliphatic carbon degradation capabilities.

### 3.5. Isolation and Screening of Antagonistic Bacteria Against Fusarium

**Figure 6:**
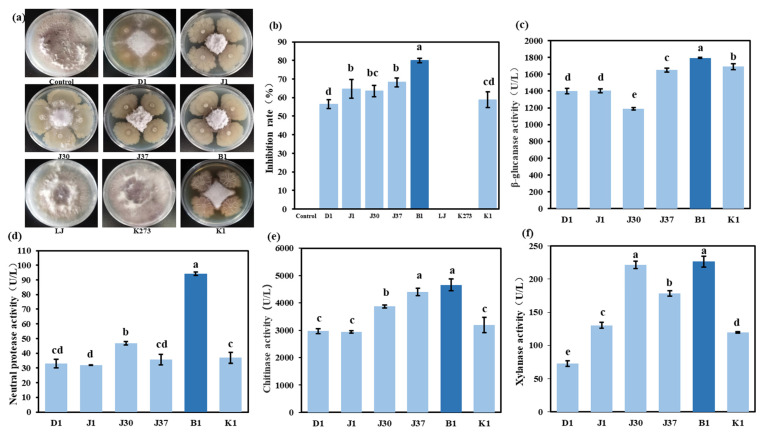
Effects of *Bacillus* strains on the growth of *F. graminearum.* (**a**) Co-culture plate diagram of Bacillus strains and *F. graminearum*. (**b**) Inhibition rate of *Bacillus* strains against *F. graminearum*. Error bars represent mean ± standard deviation. Control only inoculated with *F. graminearum*. D1, J1, J30, J37, B1, LJ, K273, and K1 are *Bacillus* strains isolated from the surface of Baudin barley. Enzyme activity of bacteria-related resistance enzymes. (**c**) β-glucanase activity of *Bacillus* strains fermentation liquid. (**d**) Neutral protease activity of *Bacillus* strains fermentation liquid. (**e**) Chitinase activity of Bacillus strains fermentation liquid. (**f**) Xylanase activity of *Bacillus* strains fermentation liquid. One-way analysis of variance was performed using SPSS 27 software. Different letters indicate significant differences between antagonistic bacteria (*p* < 0.05).

Using Baudin barley as the experimental material, a total of 30 strains of bacteria were screened on the surface of barley seeds, including eight strains of *Bacillus*. The effects of several *Bacillus* strains on *F. graminearum* are shown in Figure 6a. Among them, strains B1, J1, and J37 had advantages in inhibiting the growth of *F. graminearum* (Figure 6b).

The cell wall of *F. graminearum* is mainly composed of cellulose, chitin, β-glucan, and protein. Strain B1 had β-glucanase activity, chitinase activity, xylanase activity, and neutral protease, among which β-glucanase activity and protease activity were much higher than for other bacteria (Figure 6c–f). Therefore, strain B1 has a high ability to antagonize *Fusarium*.

### 3.6. Identification of Highly Antagonistic Bacterial Strain B1

**Figure 7:**
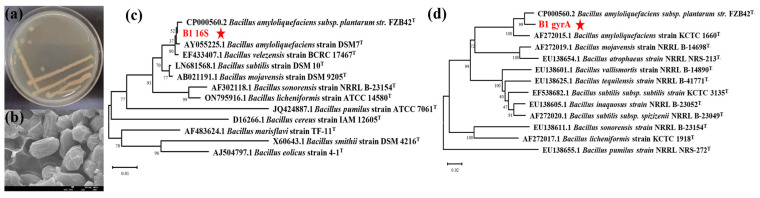
Identification of strain B1. (**a**) Colony morphology of strain B1. (**b**) SEM images of microscopic morphology of strain B1. (**c**) Phylogenetic tree based on the partial nucleotide sequence of 16S rRNA of strain B1. (**d**) Phylogenetic tree based on the partial nucleotide sequence of *gyrA* of strain B1. The phylogenetic tree was constructed by using the 16S rRNA and *gyrA* sequence of *Bacillus* species using the neighbor-joining method (using MEGA 11.0 software). The numbers at the nodes indicate the levels of bootstrap support (%) based on a neighbor-joining analysis of 1000 resampled datasets. Bootstrap values are shown on the nodes: Bars = 0.01 (**c**) and 0.02 (**d**) nucleotide substitutions per site (T = type strain).

Strain B1 was opalescent, opaque, and wrinkled on the surface with an irregular contour on the LB solid medium (Figure 7a). The bacteria were rod-shaped with no pods or flagella observed by SEM, and they were (1.20 ± 0.23) µm × (0.65 ± 0.03) µm in size, as shown in Figure 7b. Strain B1 was Gram-stain positive. The physiological and biochemical indexes of strain B1 are shown in Table 2.

The 16S rRNA (Figure 7c) and *gyrA* (Figure 7d) sequences of strain B1 were analyzed, and the phylogenetic tree was constructed. The 16S rRNA sequence of strain B1 had high homology with many types of *Bacillus* strains. It was preliminarily determined that strain B1 was *Bacillus* sp., and the *gyrA* sequence had 99% homology with the model strain of *B. amyloliquefaciens* (CP 000560.2). Strain B1 was identified as *B. amyloliquefaciens*.

### 3.7. In Vitro Inhibitory Activity of Strain B1 Against F. graminearum

**Figure 8:**
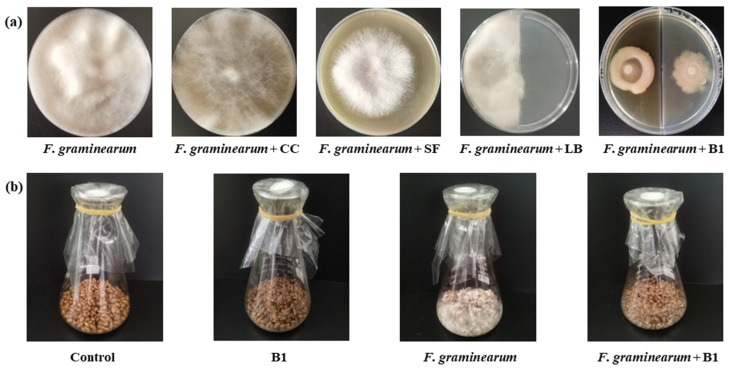
In vitro inhibition of strain B1 on *F. graminearum*. (**a**) Each component of strain B1 was co-cultured with *F. graminearum*. CC, strain B1 cell content extract; SF, strain B1 sterile fermentation liquid spread evenly on the PDA and inoculated with *F. graminearum* in the middle position; *F. graminearum* + LB, the left side was inoculated with *F. graminearum*, and the right side was inoculated with sterile LB medium; *F. graminearum* + B1, the left side was inoculated with *F. graminearum*, and the right side was inoculated with B1 fermentation liquid; all treatments cultured for 5 days. (**b**) The antibacterial effects of strain B1 on *F. graminearum* in barley medium. Control, not inoculated; B1, inoculated with B1 fermentation liquid; *F. graminearum*, inoculated with *F. graminearum*; *F. graminearum* + B1, inoculated with *F. graminearum* and B1 fermentation liquid at the same time; all treatments cultured for 7 days.

The bacteriostatic effects of SF, CC, and VOC of strain B1 are shown in Figure 8a. It can be seen that the sterile fermentation liquid and volatile compounds have bacteriostatic effects. The reported antibacterial metabolites of *B. amyloliquefaciens* are mainly proteins and lipopeptides [23], so the main antibacterial metabolites of strain B1 may be antibacterial proteins or antibacterial peptides, as well as volatile compounds.

*F. graminearum* solution was inoculated into sterilized barley to simulate the environment of barley infected by *Fusarium*. The experimental group with B1 fermentation broth added to Baudin barley showed an inhibitory effect on infection by *F. graminearum* (Figure 8b).

## 4. Discussion

*Fusarium* is one of the main pathogens of plant root rot [24]. At present, there are many reported pathogenic *Fusarium*, such as *F. graminearum*, *Fusarium oxysporum*, *Fusarium solani*, and so on [25,26,27]. Because of the environmental protection and high efficiency of biological control, biological control measures with specific bacteriostatic effects of microorganisms or their metabolites at their core have attracted more and more attention [28]. The seed microbial community is the initial source of the plant microbiota; its core bacteria have plant genotype specificity and can be stably transmitted to the next generation of plants [29,30]. The seed microbiota can more easily colonize on seedlings [31]. Through seed microbiota transplantation, this study described changes in the rhizosphere microorganisms of different seed microorganisms of the same barley after the infection by *F. graminearum*. Seed microbiota transplantation realized the conditions of different seed microbiotas on the same barley. Under these conditions, the rhizosphere microorganisms of the two barley seedlings were antagonistic to *F. graminearum*.

The effects of *F. graminearum* infection on seedling growth and community diversity of different barley cultivars were investigated. The seedling growth rates of Baudin barley and Kenpi 7 barley were inhibited after *Fusarium* infection (Figure 2). The microbial diversity of plant seeds in different growth environments responds differently to pathogenic fungi [32]. *F. graminearum* was inoculated onto barley after exchanging the microbiota on the surface of the barley seeds. The same varieties of barley, but with different seed surface microbiota were compared. For Baudin barley, barley seedlings in group A showed higher growth than those in group C. For Kenpi 7 barley, barley seedlings in group B had lower growth than those in group D (Table 1). Therefore, Baudin barley seedlings were less affected by *Fusarium* than Kenpi 7 barley seedlings due to the influence of the seed surface microbiota. The microbial community on the surface of Baudin barley has more advantages in inhibiting *F. graminearum*. Studies have found that microbial diversity is a key factor in controlling pathogen invasion of plants [30,33]. The determination of rhizosphere bacterial communities of the six groups of barley hydroponic seedlings showed that, after *F. graminearum* infection, the microbial diversity of the seed surfaces of the two barley varieties was higher than that of the control groups without *F. graminearum* infection. Among them, the bacterial microbial diversity of the Baudin barley seed surface was higher than that of Kenpi 7 barley (Figure 4c). After pathogen infection, the bacterial community abundance on the surface of the barley seeds was positively correlated with the growth index of barley seedlings (Figure 4d,e).

The effects of *Fusarium* infection on the microbiota structure of different barley varieties were investigated. Compared with the control group, *F. graminearum* infection reduced the abundance of bacterial communities in the barley rhizosphere and deteriorated the growth environment of seedlings. Compared with the experimental group C, the abundance for experimental group A was higher at the phylum and genus levels, and the growth index of the group A seedlings was also higher than that of group C. The functional abundance of rhizosphere microorganisms of the two barleys changed after the infection by *F. graminearum*, with the functional abundance related to the nitrogen cycle and carbon degradation being higher compared with the control group (Figure 5). There were some differences in the functional abundance of rhizosphere microorganisms between the two kinds of barley after infection by *F. graminearum*. The bacterial microbiota of Baudin barley promoted the metabolic pathways related to plant resilience, which was related to improved seedling health. In order to reduce the damage of pathogens to plants, the seed microbiota respond first by regulating the rapid reproduction and secretion of beneficial bacteria against antagonistic organic matter, and changing the structure and function of the microbial community [34,35,36]. Microbiota composition and changes directly or indirectly affect plant growth. The microbiota on the surface of the barley seeds can protect the plants from pathogens [31], and are an important source of *Fusarium* antagonistic bacteria.

Studies have found that in the beer brewing process, although reduced levels of fungicide residues and mycotoxins are transferred from barley and wheat to beer, some fungicide residues are still transferred from brewing components to the beer [37,38]. Therefore, it is necessary to increase the use of biological control agents and reduce fungicide residues from the source. At the same time, soybeans, peanuts, and other oil crops also have the same need to reduce fungicide residues and mycotoxins [36]. Therefore, biological control agents are superior to traditional fungicides in terms of low toxicity and low pollution, leading to urgent demand for these agents. Through the exchange of microbiotas on the surface of barley, it was concluded that the bacterial community on the surface of Baudin barley had better resistance to *F. graminearum* than Kenpi 7 barley. Furthermore, we were able to isolate the excellent *F. graminearum* antagonistic bacteria, *B. amyloliquefaciens* B1 from the surface of Baudin barley (Figure 6 and Figure 7). In recent years, studies on biological control using *B. amyloliquefaciens* have been reported globally, mainly focusing on its roles in promoting plant growth, antagonizing pathogens, and secreting antibacterial substances [39,40,41,42]. Strain B1 and its extracellular metabolites were confirmed to inhibit the growth of *F. graminearum* in preliminary in vitro experiments, but the intracellular substances of B1 did not show antibacterial activity (Figure 8). This indicates that the antibacterial ability of strain B1 mainly comes from its secreted antibacterial substances. The fermentation broth of strain B1 also showed chitinase, protease, and other resistance enzyme activities (Figure 6). These findings are similar to the characteristics of the fermentation broth of *Bacillus subtilis* LY-1, whose bacterial suspension and cell-free culture filtrate can significantly inhibit the growth of various *Fusarium* sp. [43]. It is also worth noting that in vitro antibacterial experiments also found that strain B1 can release volatile substances to inhibit the growth of *F. graminearum*. At present, many microbial volatiles have been used in the storage and preservation of food, fruits, and vegetables. Strain B1 demonstrates potential in inhibiting *F. graminearum*.

## 5. Conclusions

In conclusion, the abundance of the seed microbial bacterial community is closely related to plant growth indicators after pathogen infection. The seed microbial community diversity change is positively correlated with plant disease resistance. The seeds of different production areas have different antagonistic abilities toward pathogens. Other than soil, seeds are an important source of antagonistic bacteria. We successfully isolated *B. amyloliquefaciens* strain B1 from the surface of Baudin barley. This strain can control plant root rot by secreting antibacterial metabolites. Strain B1 and its antibacterial metabolites can be widely used in food storage and fungicides research and development.

## Figures and Tables

**Table 1:** Growth indexes of Baudin barley and Kenpi 7 barley after 10 days of hydroponics.

Varieties	Group	Root Length (cm)	Plant Height (cm)	Fresh Mass (g)	Dry Mass (g)	Root Activity (mg/g/h)
Baudin barley	CKA ^1^	4.73 ± 0.79 a ^2^	15.95 ± 1.86 a	0.31 ± 0.05 a	0.0338 ± 0.0023 a	5.27 ± 0.14 a
	A	4.38 ± 0.70 a	14.81 ± 1.19 b	0.25 ± 0.03 b	0.0325 ± 0.0013 a	4.72 ± 0.07 b
	C	2.96 ± 0.58 b	12.43 ± 1.58 c	0.20 ± 0.03 c	0.0308 ± 0.0030 a	3.77 ± 0.08 c
Kenpi 7 barley	CKB	4.52 ± 0.68 a	15.39 ± 2.29 a	0.28 ± 0.07 a	0.0332 ± 0.0019 a	4.88 ± 0.26 a
	B	3.70 ± 0.60 b	13.40 ± 2.28 b	0.23 ± 0.04 a	0.0312 ± 0.0023 a	4.18 ± 0.15 b
	D	4.02 ± 0.44 b	14.09 ± 1.48 b	0.24 ± 0.04 a	0.0322 ± 0.0028 a	4.57 ± 0.16 ab

Note: ^1^ CKA, A, C (Baudin barley): CKA—control group, not inoculated with *F. graminearum*, own microbiota; A—experimental group, inoculated with *F. graminearum*, own microbiota; C—experimental group, inoculated with *F. graminearum*, inoculated with the surface microbiota of Kenpi 7 barley after disinfection. CKB, B, D (Kenpi 7 barley): CKB—control group, not inoculated with *F. graminearum*, own microbiota; D—experimental group, inoculated with *F. graminearum*, own microbiota; D—experimental group, inoculated with *F. graminearum*, inoculated with surface microbiota of Baudin barley after disinfection. ^2^ Different letters showed that there were significant differences between different treatment groups (*p* < 0.05) of the same barley variety. Each number represents the mean ± standard deviation of three replicates.

**Table 2:** Physiological and biochemical characteristics of strain B1.

Test	Performance Characteristics	Test	Performance Characteristics
Gram stain	+ ^1^	Gelatin hydrolysis	− ^2^
Spore forming	+	Starch hydrolysis	+
Indole production	+	Citrate	+
Voges–Proskauer	+	Methyl red	−

Notes: ^1^ ‘+’ indicates a positive reaction, and ^2^ ‘−’ indicates a negative reaction.

## Data Availability

The original contributions presented in this study are included in the article. Further inquiries can be directed to the corresponding authors.

## Abbreviations

The following abbreviations are used in this manuscript:

OUTs	Operational Taxonomic Units
PDA	Potato dextrose agar
LB	Luria–Bertani
TTC	Triphenyl tetrazolium chloride
PCoA	Principal coordinate analysis
CC	Cell content extract
SF	Sterile fermentation liquid
VOC	Volatile compound
